# Long‐term intake of phenolic compounds attenuates age‐related cardiac remodeling

**DOI:** 10.1111/acel.12894

**Published:** 2019-01-24

**Authors:** Stéphanie Chacar, Joelle Hajal, Youakim Saliba, Patrick Bois, Nicolas Louka, Richard G. Maroun, Jean‐François Faivre, Nassim Fares

**Affiliations:** ^1^ Faculté de Médecine, Laboratoire de Recherche en Physiologie et Physiopathologie, LRPP, Pôle Technologie Santé Université Saint Joseph Beyrouth Liban; ^2^ Faculté des Sciences, Centre d’Analyses et de Recherche, UR GPF, Laboratoire CTA Université Saint‐Joseph Beyrouth Liban; ^3^ Laboratoire Signalisation et Transports Ioniques Membranaires (STIM) Université de Poitiers Poitiers France

**Keywords:** aging, cardiac remodeling, fibrosis, hypertrophy, oxidative stress, phenolic compounds

## Abstract

With the onset of advanced age, cardiac‐associated pathologies have increased in prevalence. The hallmarks of cardiac aging include cardiomyocyte senescence, fibroblast proliferation, inflammation, and hypertrophy. The imbalance between levels of reactive oxygen species (ROS) and antioxidant enzymes is greatly enhanced in aging cells, promoting cardiac remodeling. In this work, we studied the long‐term impact of phenolic compounds (PC) on age‐associated cardiac remodeling. Three‐month‐old Wistar rats were treated for 14 months till middle‐age with either 2.5, 5, 10, or 20 mg kg^−1^ day^−1^ of PC. PC treatment showed a dose‐dependent preservation of cardiac ejection fraction and fractional shortening as well as decreased hypertrophy reflected by left ventricular chamber diameter and posterior wall thickness as compared to untreated middle‐aged control animals. Analyses of proteins from cardiac tissue showed that PC attenuated several hypertrophic pathways including calcineurin/nuclear factor of activated T cells (NFATc3), calcium/calmodulin‐dependent kinase II (CAMKII), extracellular regulated kinase 1/2 (ERK1/2), and glycogen synthase kinase 3ß (GSK 3ß). PC‐treated groups exhibited reduced plasma inflammatory and fibrotic markers and revealed as well ameliorated extracellular matrix remodeling and interstitial inflammation by a downregulated p38 pathway. Myocardia from PC‐treated middle‐aged rats presented less fibrosis with suppression of profibrotic transforming growth factor‐ß1 (TGF‐ß1) Smad pathway. Additionally, reduction of apoptosis and oxidative damage in the PC‐treated groups was reflected by elevated antioxidant enzymes and reduced RNA/DNA damage markers. Our findings pinpoint that a daily consumption of phenolic compounds could preserve the heart from the detrimental effects of aging storm.

## 1 INTRODUCTION

Aging is a physiological process that affects the overall health status of the organism. As the average life expectancy continues to rise in the developed world, aging has been the world's biggest killers for the last 15 years (WHO, 2017). Aging by itself is a leading risk factor for the development of several diseases, including cardiovascular ones (Liao et al., 2015). Both heart and vessels are subject to significant phenotypic and genotypic remodeling, leading to cardiovascular function impairment. At a more elaborate level, degeneration of the intracellular defense mechanisms with age stands behind major structural and functional changes of the heart (Steenman & Lande, 2017). In other words, the disruption of homeostasis between reactive oxygen species (ROS) and endogenous antioxidants leads to oxidative stress. This process is associated with lipid peroxidation, DNA damage, cellular dysfunction, and induction of apoptosis (Azevedo, Polegato, Minicucci, Paiva, & Zornoff, 2016). It interferes with the modulation of several intracellular signaling pathways involved in all aspects of cardiac fibrosis, inflammation, and hypertrophy, and, therefore, notably promoting the pathogenesis of cardiovascular diseases (Kubin et al., 2011; Moris et al., 2017; Zhao et al., 2015).

Oxidative stress appears to be a crucial modulator of the age‐related fibrotic process, in which, an excessive appearance of extracellular matrix (ECM) contributes to tissue injury (Richter & Kietzmann, 2016). The prevalence of cardiac interstitial and perivascular fibrosis has been repeatedly reported in the senescent heart and has been associated with elevated levels of ROS, endothelin‐1, total collagen content, and profibrotic transforming growth factor (TGF)‐β (Biernacka & Frangogiannis, 2011).

Progressive cardiac fibrosis is nowadays considered as a contributing factor leading to ventricular stiffness and cardiac dysfunction (Gyongyosi et al., 2017; Horn & Trafford, 2016).

Extensive evidence suggests that aging also induces an inflammatory response in the host's heart and that oxidative stress and inflammation are interdependent mechanisms (Petersen & Smith, 2016). In fact, repeated exposure to ROS causes oxidative cell damage that may contribute to a proinflammatory signaling response such as the release of tumor necrosis factor alpha (TNF‐ɑ). Moreover, the binding of this proinflammatory cytokine to its receptor activates the nuclear factor‐kB (NF‐kB) inflammasome, therefore promoting the production of interleukin‐1β (IL‐1β) among many others (Kim et al., 2008). Importantly, TNF‐ɑ, C‐reactive protein (CRP), NF‐kB, and cyclooxygenase 2 (COX‐2) are the major inflammatory mediators in the heart (Lopez‐Candales, Hernandez, Hernandez‐Suarez, & Harris, 2017).

As for cardiac hypertrophy, a form of structural and functional remodeling of the heart, it could normally occur in response to increases of ROS during aging (Martin‐Fernandez & Gredilla, 2016). ROS can directly or indirectly induce hypertrophy through activation of several intracellular proteins and signaling pathways such as mitogen‐activated protein kinase (MAPK), protein kinase C (PKC), NF‐kB, calcineurin, and tyrosine kinases (Heineke & Molkentin, 2006; Moris et al., 2017).

Aging is an inevitable physiological process, but the associated complications could be avoided according to the individual lifestyle. Before reaching a level where a medicinal treatment becomes necessary to heal the resultant complications of aging, the prevention of fibrosis, hypertrophy, and inflammation that occur in response to oxidative stress has been hypothesized through a supplementation with nutrients or an antioxidant‐rich diet such as polyphenol‐rich matrices, based on other nonaging cardiac stress animal models (Khurana, Venkataraman, Hollingsworth, Piche, & Tai, 2013).

Phenolic compounds (PC) exert several medicinal and health benefits (Varzakas, Zakynthinos, & Verpoort, 2016). Studies have shown that PC possess physiological properties favoring antidiabetes, vasodilatory, antimicrobial, antithrombotic, antiinflammatory, antihypertensive, and especially antioxidant effects (Conforti et al., 2008; Dai, Chen, Johnson, Szeto, & Rabinovitch, 2012; Hanhineva et al., 2010; Khurana et al., 2013). These compounds have been speculated to delay aging through mechanisms that involve the reduction of ROS. However, the long‐term in vivo use of these molecules as a mixture which reflects their daily consumption in a food matrix has not been investigated yet. In this study, the long‐term cardiac impact of a daily consumption of different concentrations of a PC mixture during the lifespan of healthy rats was explored. During the treatment, hemodynamic and functional cardiovascular parameters were evaluated in all groups. At 17 months old, approximately at their middle‐age, cardiac hypertrophic, fibrotic, inflammatory, and apoptotic and oxidative signaling pathways were investigated.

## 2 RESULTS

### 2.1 Phenolic compounds preserve cardiac morphological and functional properties altered with aging

**Figure 1:**
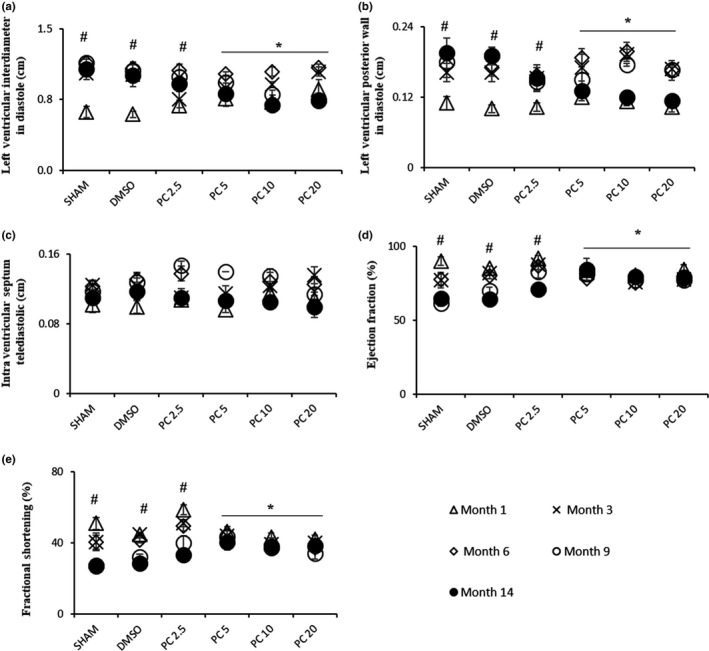
Dose‐dependent recovery by phenolic compounds of age‐related hypertrophy observed by echocardiography. Left ventricular chamber and cardiac performance were over 14 months with or without PC or DMSO. Results are expressed as mean ± *SEM*. (a) LVIDd, (b) LVPWd, (c) IVSTd, (d) EF, and (e) FS. For SHAM‐DMSO rats: *n* = 6/group, and treated rats with PC: *n* = 8/group. #*p* < 0.05, time effect within each group (Month 14 vs. Month 1). *p < 0.05, effect of treatment after 14 months vs. SHAM and DMSO). PC 2.5 = phenolic compounds at 2.5 mg kg^−1^ day^−1^; PC 5 = phenolic compounds at 5 mg kg^−1^ day^−1^; PC 10 = phenolic compounds at 10 mg kg^−1^ day^−1^; PC 20 = phenolic compounds at 20 mg kg^−1^ day^−1^

Given that cardiac structure and performance are subjected to changes with age (Lakatta & Levy, 2003), we investigated by echocardiography the heart status in our model. Rats from all groups exhibited comparable cardiac chamber morphology and contractility at baseline (Figure 1). At the age of 17 months, the left ventricular internal diameter in diastole (LVIDd) and the left ventricular posterior wall in diastole (LVPWd) were significantly higher in the control groups (SHAM and DMSO) compared to baseline values (Figure 1a,b). In contrast, no significant effect of time or treatment was observed on end‐diastolic interventricular septum (IVSTd) in all groups (Figure 1c). Interestingly, LVPWd and heart chamber volume were rescued in groups treated with PC >2.5 mg/kg after 14 months of treatment. On the other hand, ejection fraction (EF) and fractional shortening (FS) of control rats were significantly depressed with age when compared to baseline values (Figure 1d,e). However, following 14 months of PC 5, PC 10, and PC 20 consumption, cardiac function was ameliorated, and EF/FS values were significantly higher vs. the control groups (Figure 1 d,e).

Body weights (BW), whole heart weights (HW), and tibia lengths (TL) of all rats were also measured. Table 1 shows that HW/BW ratio remained unchanged during aging. However, HW/TL ratio increased significantly in the control and PC‐treated groups compared to young rats.

**Table 1:** Characteristics of aged rat hearts in the control and treated groups

	Young	SHAM	DMSO	PC 2.5	PC 5	PC 10	PC 20
HW/BW (mg/g)	2.74 ± 0.12	2.73 ± 0.07	2.74 ± 0.09	2.9 ± 0.1	3.05 ± 0.04	2.85 ± 0.15	2.75 ± 0.07
HW/TL (mg/mm)	22.08 ± 1.49	32.31 ± 0.77*	31.3 ± 1.17*	35.33 ± 2.09*	35.79 ± 1.66*	35.5 ± 1.38*	33.54 ± 1.17*

Note

Values are means ± *SEM*.

BW: body weight; HW: heart weight; TL: tibia length.

* *p* < 0.001 compared to the young group.

### 2.2 Long‐term consumption of PC attenuates age‐induced cardiac hypertrophy

**Figure 2:**
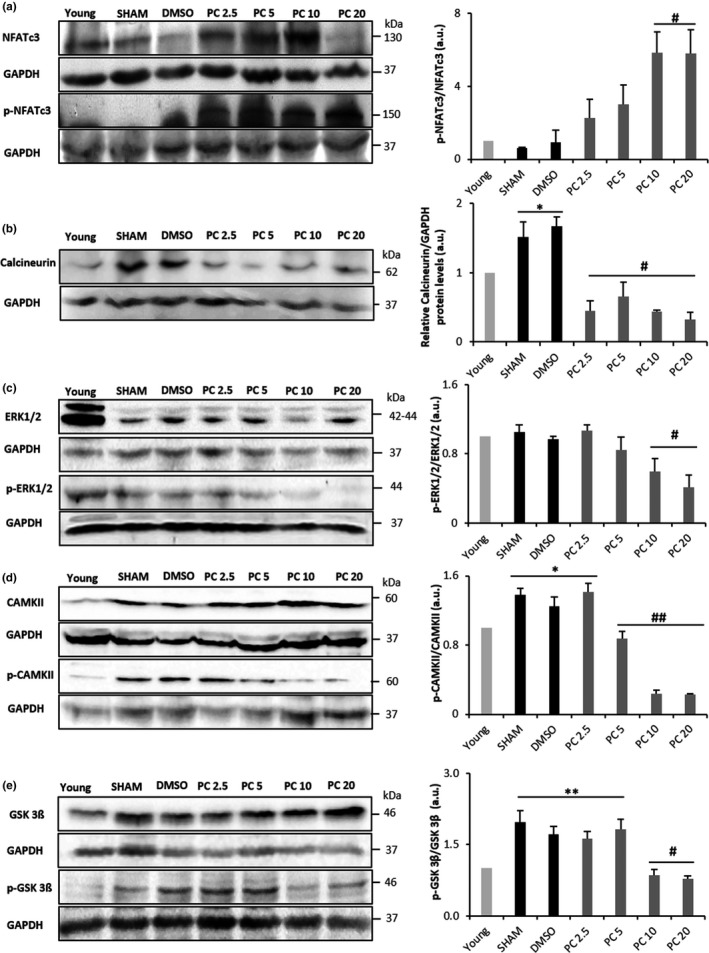
Modulation of age‐induced cardiac remodeling by phenolic compound consumption. Representative western blot (left) and histograms analyses (right) for NFAT (a), calcineurin (b), ERK1/2 (c), CAMKII (d), and GSK 3ß (e) in rat cardiac tissue, normalized to GAPDH (in arbitrary units, a.u.). Values represent mean ± *SEM* (*n* = 3 for each protein and condition). *p < 0.05 and **p < 0.01 vs. young, #*p < *0.05 and ##*p < *0.01 vs. SHAM and DMSO. PC x = phenolic compounds at x mg kg^−1^ day^−1^

Aging induces changes in the expression of proteins involved in the cellular hypertrophic pathways such as NFATc3, calcineurin, ERK1/2, CAMKII, and GSK 3ß (Mudd & Kass, 2008). In the present study, aging resulted in the increased expression of calcineurin (Figure 2b) and the phosphorylated‐to‐total levels ratio of CAMKII (Figure 2d) and GSK 3ß (Figure 2e). Interestingly, these effects were no more observed when rats were treated with PC which seem to dose‐dependently and completely abolish them. Moreover, NFATc3 (Figure 2a) and ERK1/2 (Figure 2c), which expression levels were not altered in our aging model, presented significantly modified phosphorylation levels in high‐dose PC‐treated groups. In order to check whether control rat hearts are in a condition of severe disease, cardiac stress markers, brain natriuretic peptide (BNP), and troponin were measured. No significant differences were observed for BNP plasma levels and troponin expression in all groups (Supporting Information Figure S1a,b).

### 2.3 Chronic PC treatment discharges heart from age‐associated inflammation impact

**Figure 3:**
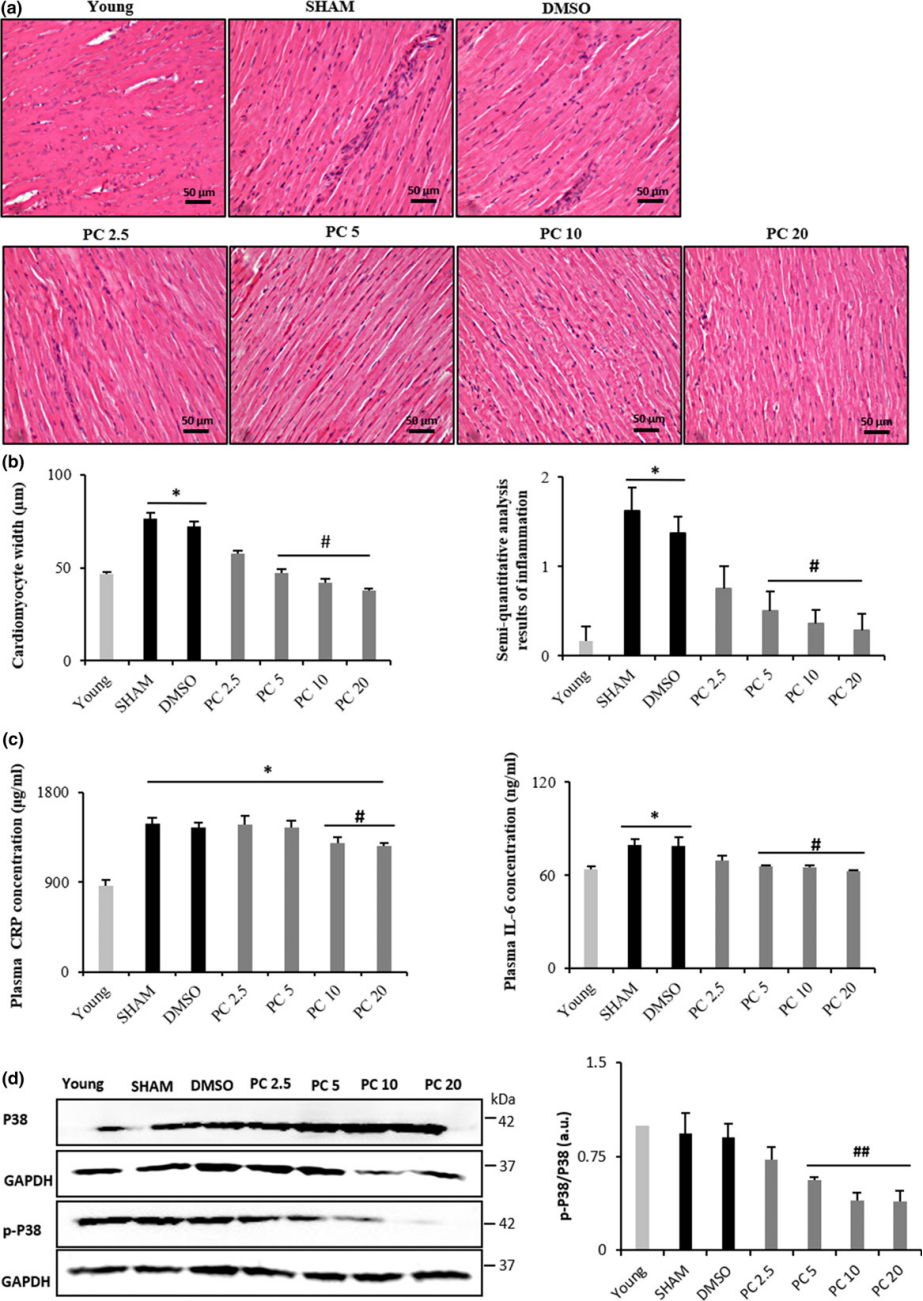
Dose‐dependent reduction of age‐related cardiac inflammation by phenolic compounds. (a) Representative microphotographs of left ventricular sections of the heart stained with hematoxylin‐eosin showing young, SHAM, DMSO, PC 2.5, PC 5, PC 10, and PC 20 groups. (b) Histograms showing cardiomyocytes width (μm) and semiquantitative scores of inflammation (*n* = 6–8 per group). (c) CRP and IL‐6 plasma concentrations in all groups (*n* = 6–8 per group). (d) Representative western blot analyses for p38 in rat cardiac tissue, normalized to GAPDH (in arbitrary units, a.u.) (*n* = 3 for each protein and condition). *p < 0.05 vs. young, #*p < *0.05 and ##*p < *0.01 vs. SHAM and DMSO. Results are mean ± *SEM*. PC x = phenolic compounds at x mg kg^−1^ day^−1^

Inflammation is a feature commonly observed in the aged heart (Wu, Xia, Kalionis, Wan, & Sun, 2014). Accordingly, semiquantitative analysis of myocardial sections stained with hematoxylin‐eosin revealed cardiomyocyte hypertrophy and interstitial inflammation in 17‐month‐old control rats. Interestingly, PC induced a dose‐dependent and significant impeding of these age‐related processes (Figure 3a,b). This was accompanied by a significant decrease of CRP and IL‐6 plasma concentrations (Figure 3c), and a significant downregulation of P38 phosphorylation level (Figure 3d).

### 2.4 Long‐term consumption of PC protects the heart from fibrogenesis occurring with age

**Figure 4:**
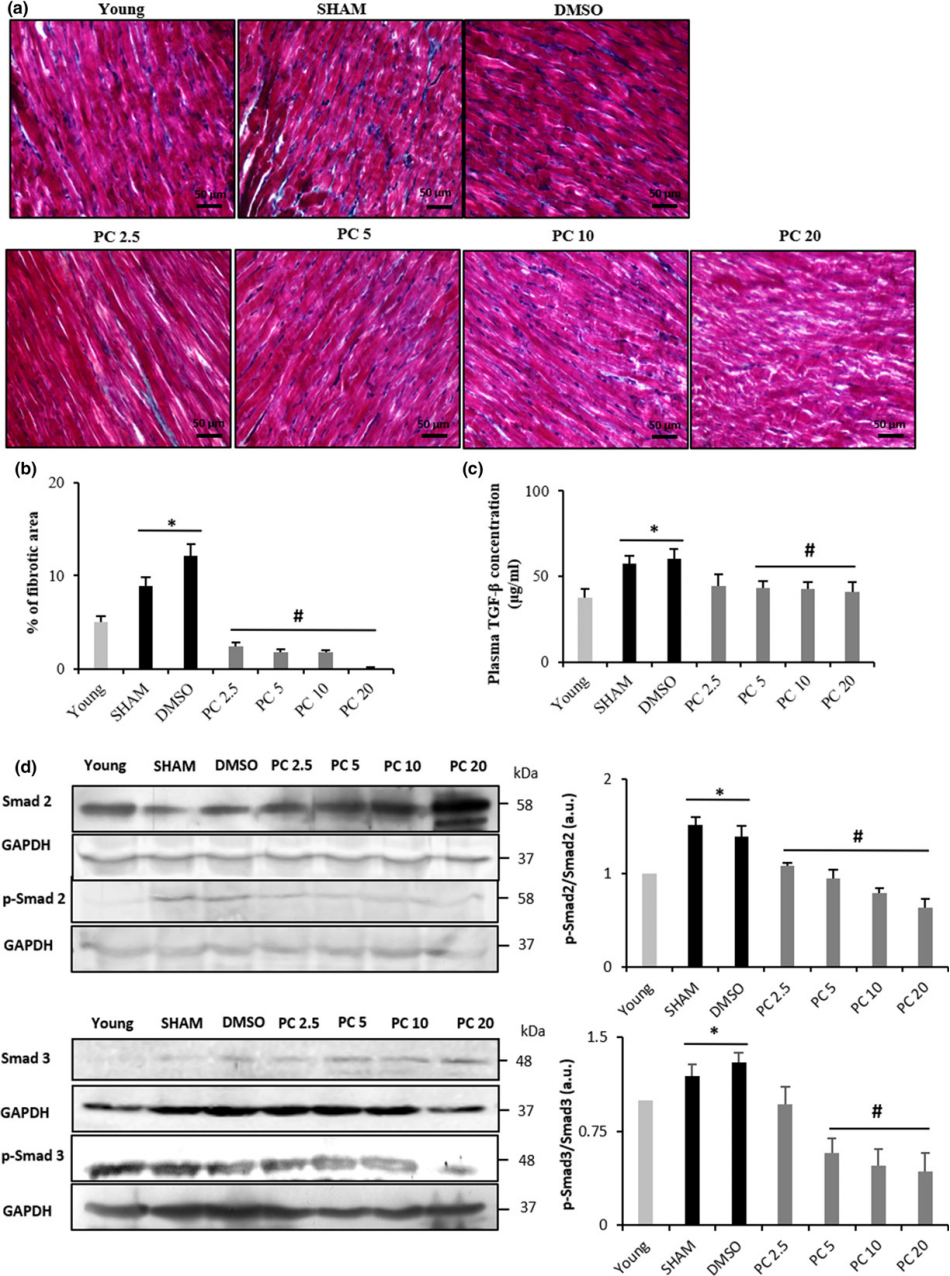
Dose‐dependent regression of fibrosis related to advanced age by phenolic compound consumption. Quantification of interstitial fibrosis by Masson's trichrome staining of the left ventricle from SHAM, DMSO, and PC‐treated rats. (a) Collagen accumulation is shown in blue color. The control rat hearts (SHAM and DMSO) show increased interstitial fibrosis compared to the PC rat hearts. (b) Histogram showing percentage of fibrotic area (*n* = 6–8 per group). (c) TGF‐ß plasma concentrations in all groups (*n* = 6–8 per group). (d) Representative western blot (left) and histograms analyses (right) for Smad2 and Smad3 (*n* = 3 for each protein and condition) in rat cardiac tissue, normalized to GAPDH (in arbitrary units, a.u.). *p < 0.05 vs. young and #*p < *0.05 vs. SHAM and DMSO. Results are mean ± *SEM*. PC x = phenolic compounds at x mg kg^−1^ day^−1^

With the onset of advanced age, the heart becomes vulnerable to fibrosis (Biernacka & Frangogiannis, 2011). Accordingly, the percentage of fibrotic area in hearts of 17‐month‐old control groups was significantly higher than in young (Figure 4a,b). This was evidenced by semiquantitative analysis of myocardial sections stained with Masson's trichrome. PC drastically impeded this age‐related fibrosis, even at the lowest tested concentration. In view of this, we assessed TGF‐β/Smad signaling pathway. TGF‐β plasma concentration was increased in the control groups compared to young rats, an effect which was significantly inhibited in the PC‐treated groups (Figure 4c).

These changes were further confirmed with assessment of cellular Smad2 and Smad3 imbalance. Figure 4d shows a significant increase in phospho‐Smad2/Smad2 ratio in aged control groups vs. young, an effect that was not present in 17‐month‐old PC‐treated rats. As for Smad3, phospho‐Smad3/Smad3 ratio underwent the same imbalance as compared to young and PC‐treated groups. (Figure 4d).

### 2.5 Chronic PC treatment immunes the heart from oxidative stress

**Figure 5:**
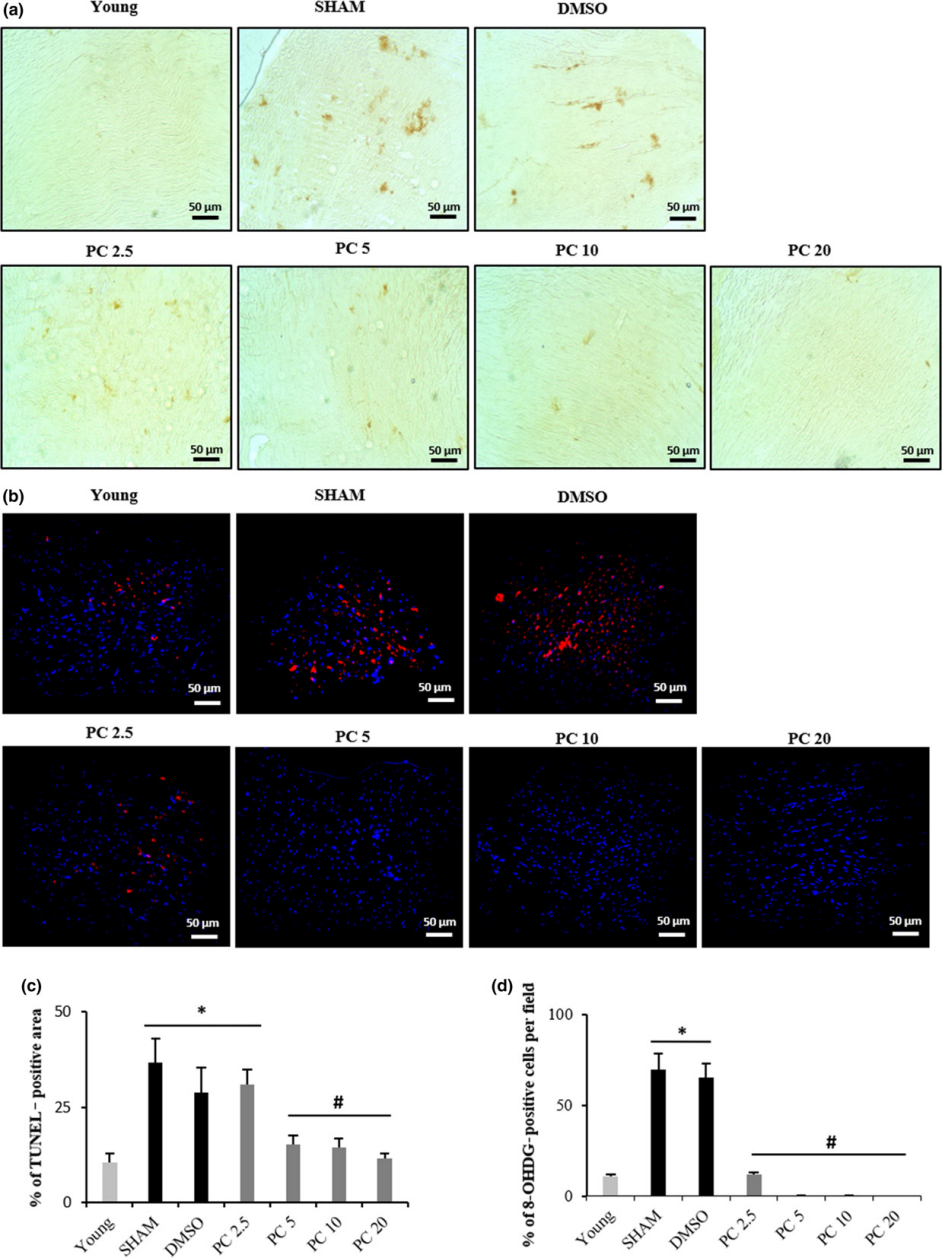
Dose‐dependent inhibition of apoptosis and DNA/RNA oxidative damage in PC‐treated rat cardiac tissues. Representative images of TUNEL (a) and 8‐OHdG immunohistochemistry (b) in hearts obtained from young, SHAM, DMSO, PC 2.5, PC 5, PC 10, and PC 20 groups. Dark brown stains in (a) represent apoptotic nuclei. (c) Quantification of percentage of TUNEL‐positive areas. (d) % of 8‐OHDG‐positive cells. *n* = 6–8 per group. Values are mean ± *SEM*. *p < 0.05 and #*p < *0.05 represent the comparison between all groups. PC x = phenolic compounds at x mg/kg/day

**Figure 6:**
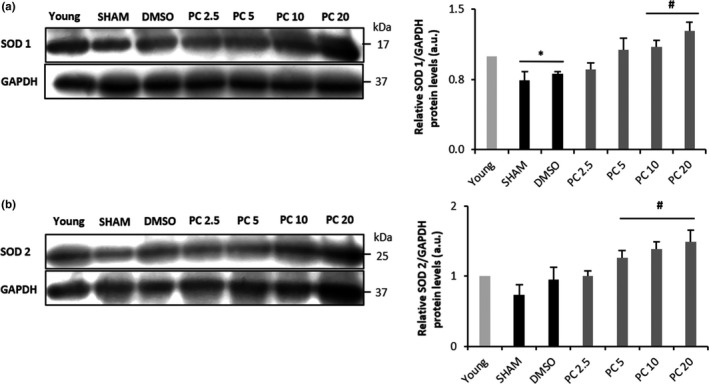
Dose‐dependent restoration by phenolic compounds of reduced activity of (SOD) 1 and 2 in SHAM, DMSO, and PC2.5. Western blot detection of SOD1 and SOD2 from cardiac tissue of control and PC‐treated rats, normalized to GAPDH (in arbitrary units, a.u.) (*n* = 3 for each protein and condition). *p < 0.05 vs. young and #*p < *0.05 vs. SHAM and DMSO

Cellular aging is characterized by increased ROS production, apoptosis, and the accumulation of damaged proteins and organelles (Storz, 2006). Thus, we carried out TUNEL staining, that is, terminal deoxynucleotidyl transferase‐mediated dUTP nick end‐labeling, to ascertain the extent of apoptotic cells in 17‐month‐old hearts of control and PC‐treated rats. The percentage of TUNEL‐positive cells in control rats was significantly higher than that of young. PC dose‐dependently impeded this age‐related increase, with no effect at 2.5 mg/kg but with significant reductions at 5, 10, and 20 mg/kg (Figure 5a,c). Immunohistochemical assessment for 8‐OHdG, a critical biomarker of DNA damage (Valavanidis, Vlachogianni, & Fiotakis, 2009), was also conducted to demonstrate its abundance in aging cardiac tissues and the inhibitory effect of PC. Indeed, increased immunostaining for 8‐OHdG was evident in the control groups vs. PC‐treated rats (Figure 5b,d). Finally, expression of the antioxidant enzyme SOD1 was reduced in aged hearts, an effect no longer observed in the presence of PC at concentrations higher than 2.5 mg/kg (Figure 6a). Similarly, expression of the antioxidant enzyme SOD2 was significantly higher in PC5, PC 10, and PC 20 vs. the control groups (Figure 6b).

## 3 DISCUSSION

Aging is a physiological process that affects the overall health status of the organism and is a leading risk factor for the development of cardiovascular diseases. Several studies have shown the beneficial effects of specific individual PC consumption on the heart (Rasines‐Perea & Teissedre, 2017); however, no studies to our knowledge have evaluated the in vivo cardiac effects of chronic dietary doses of a PC mixture. Therefore, the aim of this work was to evaluate, for the first time, the long‐term in vivo impact of a mixture of PC on age‐associated cardiac remodeling.

Long‐term consumption of PC preserved cardiac morphological and functional properties. At 17 months old, when compared to young rats, the control groups revealed a marked increase in heart chamber and LVPWd, an effect which was not observed in the PC‐treated groups (PC >2.5 mg/kg). Cardiac chamber remodeling is features of cardiac senescence within age (Lakatta & Levy, 2003). Some PC such as resveratrol and the synthetic flavonoid derivative S17834 have been demonstrated to exhibit beneficial effects against the development of cardiac chamber remodeling but in specific pathological conditions not related to aging (Qin et al., 2012; Thandapilly et al., 2010). Cardiac aging is also characterized by the decline of EF and FS (Boluyt, Converso, Hwang, Mikkor, & Russell, 2004). We confirmed in our study this age effect on cardiac performance, while no significant decrement was observed in the PC‐treated groups (PC >2.5 mg/kg). In other words, it seems that daily consumption of PC was able to protect the heart not only against age‐related remodeling, but also against age‐related decrease in cardiac functional performance.

Previous works showed an enhancement of cardiac hypertrophic signaling pathways in the aged heart, specifically, NFATc3/calcineurin (Dai et al., 2009), ERK1/2 (Liao et al., 2015), CAMKII (Nattel, 2018), and GSK 3ß (Fallah, Chelvarajan, Garvy, & Bondada, 2011). Altogether, dephosphorylation of NFAT by calcineurin and phosphorylation of GSK 3ß, CAMKII, and ERK1/2 coordinate the cardiac hypertrophic response (Heineke & Molkentin, 2006). These effects were confirmed in our model. Interestingly, stimulation of these pathways is not necessarily associated with an increase in heart mass, as shown in our model where HW/TL ratio remained unchanged with age, but may depend on a balance between progressive myocyte dropout in aging and reactive myocyte hypertrophy accompanied with an increased collagen concentration (Anversa, Hiler, Ricci, Guideri, & Olivetti, 1986; Olivetti, Melissari, Capasso, & Anversa, 1991). Dolinsky et al. (2015) demonstrated that resveratrol was able to prevent stress‐induced cardiac hypertrophy, but no studies have been undertaken on the long‐term effect of dietary doses of PC on age‐induced cardiac hypertrophy. Our results clearly show that PC concentrations were able to dose‐dependently and significantly attenuate the age effect on key signaling regulators of cardiac hypertrophy.

Our findings also show a significant increase of plasma CRP and IL‐6 as well as leukocyte infiltrates in the myocardium of control rats compared to PC‐treated ones, suggesting that chronic PC treatment discharged the heart from age‐associated inflammation impact. CRP and IL‐6 are plasma inflammatory markers that increase with aging heart (Franceschi & Campisi, 2014). Usually, their secretion could be predictive of subsequent cardiovascular events such as acute coronary syndrome (Wang, Liu, Wang, & Jin, 2014). It has been demonstrated that resveratrol suppresses the secretion of proinflammatory cytokines in murine macrophages by modulating NF‐κB signaling pathway (Ma, Wang, Dong, Li, & Cai, 2015). Additionally, P38 kinase plays a central role in inflammation activated by stress and inflammatory cytokines (Roux & Blenis, 2004). Herein, the P38 inflammatory pathway was upregulated in rat hearts conversely to that observed in the PC‐treated groups (PC5, PC 10, and PC 20). This corroborated the results of histology and plasma inflammatory markers.

PC mixture consumption protected the heart from fibrogenesis in PC‐treated rats. Indeed, both, Smad2 and Smad3 activities were reduced in the myocardium, as well as TGF‐ß plasma concentration. Furthermore, less percentage of fibrotic area was detected in the PC‐treated myocardia compared to controls. Aging increases TGF‐ß expression, which plays an important role in the synthesis and secretion of collagen by cardiac fibroblasts, thus contributing to cardiac stiffening (Nakou et al., 2016). Kaempferol and PC of a yacon plant were shown to alleviate, respectively, myocardial fibrosis in angiotensin II‐induced cardiac dysfunction and in diabetic rat model (Dos Santos et al., 2018; Liu et al., 2017). Nevertheless, these experiments were undergone in pathological conditions not related to normal aging. In the present work, we reveal that long‐term PC consumption impeded the impact of aging process on cardiac fibrosis in a dose‐dependent manner.

Finally, concomitant with fibrosis, cellular aging is characterized by increased ROS production, apoptosis, and accumulation of damaged proteins and organelles (Storz, 2006). In the present work, apoptotic myocytes were remarkably reduced in the PC‐treated groups when compared to control, with an increased expression of antioxidant enzymes SOD 1 and SOD 2. Long‐term oxidative stress has been linked to various diseases, especially cardiovascular ones (Petersen & Smith, 2016). The mechanism of cardiac hypertrophy prevention by flavonoids has been suggested to be related to the regression of cardiomyocyte apoptosis and oxidative stress (Sheng, Gu, Xie, Zhou, & Guo, 2009). In our model, the observed beneficial effects of PC on age‐induced cardiac remodeling might be likely due to their antioxidant properties. Enhancement in SOD 1 and SOD 2 counterbalances ROS production in cardiac tissue and therefore abrogates myocardial injuries.

In conclusion, our findings pinpoint for the first time that a long‐term daily consumption of PC preserves cardiac morphology and performance with less hypertrophy, inflammation, fibrosis, and cardiomyocyte apoptosis. These results might propose an effective nutritional intervention for cardiac protection during aging.

## 4 EXPERIMENTAL PROCEDURES

### 4.1 Characterization of phenolic compound mixture

The grape pomace by‐products used in this work are the solid remains of grapes obtained in wine industries after the pressing step. They contain the skins, pulp, seeds, and stems of the fruit. We used three different grape types: Cabernet Sauvignon, Marselan, and Syrah. The corresponding by‐products were stored at −20°C until use. Once defrosted at room temperature, PC extraction and characterization were performed as previously described (Chacar et al., 2018). Briefly, the PC were extracted from grape pomace extracts via a solid–liquid extraction, and then, the solutions were spray‐dried and we obtained a powder consisting of a pure mixture of PC. Total PC contained in the powder were quantified using the Folin–Ciocalteu colorimetric assay against a standard curve of gallic acid and expressed as mg of gallic acid equivalent (GAE)/g of the final powder (Singleton, Orthofer, & Lamuela‐Raventos, 1999). The free radical scavenging capacity (AC) of the powder was measured by means of the DPPH assay (Brand‐Williams, Cuvelier, & Berset, 1995). The DPPH value of the extracts was expressed in micromolar Trolox equivalent per milliliter (µMTE). High‐performance liquid chromatography (HPLC) analyses were conducted for the identification and quantification of PC from grape pomace extracts. The HPLC‐DAD analyses were carried out with an HPLC system (Waters Alliance, USA) equipped with a quaternary Waters e2695 pump. The method was adapted based on the review by Khoddami, Wilkes, and Roberts (2013). The results of Folin–Ciocalteu analysis showed that the final powder contains 920 mg/g of PC (92% of the total mass of the powder). The left 8% are residual sugars and other not determined compounds coextracted with PCs. High free radical scavenging capacity of grape pomace extract consists of 6,028 µMTE equivalent to 92% of inhibition of free radicals. As for the HPLC results, eight compounds were detected (malvidin: 42 mg/g of powder; delphinidin: 27.8; rutin hydrate: 5.8; quercetin: 3.4; catechin: 0.8; coumaric acid: 0.75; kaempferol: 0.39; and trans‐cinnamic acid: 0.17).

### 4.2 Animals and diets

The present study was approved by the Ethical Committee of the Saint Joseph University of Beirut. The protocols were designed according to the Guiding Principles in the Care and Use of Animals approved by the Council of the American Physiological Society and were in adherence to the *Guide for the Care and Use of Laboratory Animals* published by the US National Institutes of Health (NIH Publication no. 85‐23, revised 1996) and according to the European Parliament Directive 2010/63 EU. Forty‐four male adult Wistar rats were used. The animals were kept at a stable temperature (22 ± 3°C) and humidity (50% ± 5%) and were exposed to 12:12 hr light–dark cycle. They were fed ordinary rodent chow and were acclimatized at least 1 week under these conditions before the start of the study. The treatment was conducted for 14 months. The rats were divided into six groups randomly: four treated groups (*n* = 8 per group) with different concentrations of PC (2.5 mg kg^−1^ day^−1^, 5 mg kg^−1^ day^−1^, 10 mg kg^−1^ day^−1^, and 20 mg kg^−1^ day^−1^ diluted in the drinking water plus 0.1% DMSO); one group SHAM (*n* = 6) and one group control (0.1% DMSO, *n* = 6). The concentrations of PC were calculated and administered to the rats following the quantification of these compounds found in the powder as obtained by Folin–Ciocalteu method in order to obtain the exact final amount 2.5, 5, 10, and 20 mg kg^−1^ day^−1^. Adjustments of the PC doses were made every one month according to the rat weights, in order to maintain a stable intake.

### 4.3 Transthoracic echocardiography

Transthoracic echocardiography was performed every 3 months using the SonoScape S2V high‐resolution color Doppler ultrasound system equipped with a 10 MHz L741 probe (SonoScape Co., Shenzhen, China). Rats were sedated by intraperitoneal injection with ketamine (Ilium, Australia; 75 mg/kg) and xylazine (Interchemie, Holland; 10 mg/kg). Left ventricular (LV) parasternal long‐axis 2D view in *M*‐mode was performed at the level of papillary muscle to assess LV wall thicknesses and internal diameters, allowing the calculation of the fractional shortening (FS) and ejection fraction (EF) by the Teicholz method. EF (%) was calculated using the following formula: EF = (EDV − ESV)/EDV × 100; EDV: end‐diastolic volume and ESV: end‐systolic volume. FS (%) was calculated based on the diameters of the left ventricle: FS = (LVIDd − LVIDs)/LVIDd × 100.

### 4.4 Histological analyses

For sacrifice, animals were anesthetized with the same ketamine/xylazine mixture and pedal withdrawal reflex was performed to make sure of adequate depth of anesthesia. When animals were completely nonresponsive to toe pinching, their hearts were removed, weighted, and perfused with ice‐cold Tyrode solution until all blood was removed and then cut into half through a mid‐sagittal plane. One half was kept at −80°C for protein extraction, whereas the other half fixed in 10% formalin solution (Sigma‐Aldrich, St. Louis, MO, USA). The formalin‐fixed tissue was embedded in paraffin, and sections of 5 μm thickness were cut. Paraffin‐embedded sections of the hearts were stained with either hematoxylin‐eosin or Masson's trichrome for histopathological evaluation (Sigma‐Aldrich, St. Louis, MO, USA). After staining, the sections were rinsed in distilled water, dehydrated in ethanol/water baths with decreasing water content, and finally rinsed in xylene before being mounted with a permanent mounting medium. Histological examinations were performed by two independent pathologists blinded to the conditions. Six or eight sections were analyzed for each group. A semiquantitative scoring system was used to assess interstitial changes such as inflammation and fibrosis. Furthermore, RNA/DNA oxidative damage assay was conducted using immunofluorescence on heart sections with an antibody (8‐OHDG) that recognizes 8‐hydroxy‐2’‐deoxyguanosine, 8‐oxo7,8‐dihydroguanine, and 8‐oxo‐7,8‐dihydroguanosine (Abcam, Cambridge, UK). Staining was performed after triton permeabilization and saturation with goat serum and bovine serum albumin. Nuclei were costained with 4′,6‐diamidino‐2‐phenylindole (DAPI). In situ apoptosis detection kit (Abcam) was also used to recognize apoptotic nuclei in heart sections. Fibrosis and apoptosis analyses were done with the ImageJ program, by thresholding the acquired pictures, then creating selections of the fibrotic and apoptotic areas; 8‐OHDG/DAPI‐positive cells were also counted.

### 4.5 Protein measurement and western blotting

Protein concentrations were measured by the Bradford method using bovine serum albumin as standard (Bio‐Rad, USA). Protein samples (100 μg) were separated in a vertical electrophoresis system that worked at 100 volts (V). Once the electrophoresis was completed, the gel was removed and then transferred to polyvinylidene fluoride (PVDF) membrane (Bio‐Rad Laboratories Inc., Irvine, CA, USA) under electrophoretic conditions (80 V, 400 mA, 2h30). Membranes were blocked for 1 hr at room temperature in TBS‐Tween blocking solution (in mmol/l: Tris‐HCl 100, NaCl 150, and Tween‐20 0.1%) with 5% BSA or 5% nonfat dry milk. The blots were then incubated overnight with gentle agitation at 4°C with primary antibodies: NFATc3 (Santa‐Cruz Biotechnology, Dallas, TX, USA; 1/500), phospho‐Ser165 NFATc3 (p‐NFATc3) (Abcam; 1/500), PP2B‐Aβ (C‐20) (Santa‐Cruz Biotechnology; 1/1,000), SOD1 (Abcam; 1/2000), SOD2 (Abcam; 1/4,000), Smad2 (Abcam; 1/500), phospho‐S467 Smad2 (p‐Smad2) (Abcam; 1/500), Smad3 (Abcam; 1/1,000), phospho‐S423+S425 Smad3 (p‐Smad3) (Abcam; 1/1,000), pan‐CAMKII (Cell Signaling Technology, MA, USA; 1/1,000), pan‐phospho CAMKII (Cell Signaling Technology; 1/1,000), troponin I (C‐4) (Santa‐Cruz Biotechnology; 1/500), GSK 3β (Cell Signaling Technology; 1/1,000), phospho‐GSK 3β (Cell Signaling Technology; 1/1,000), ERK1/2 (Abcam; 1/1,000), phospho‐ERK 1/2 (Abcam; 1/10,000), P38 (Cell Signaling Technology; 1/1,000), phospho‐Thr180/Tyr182 P38 (Cell Signaling Technology; 1/1,000), NF‐ƙB p65 (Ser536) (Cell Signaling Technology; 1/1,000), phospho‐NF‐ƙB p65 (Ser536) (93H1) (Cell Signaling Technology; 1/1,000), and glyceraldehyde‐3‐phosphate dehydrogenase (GAPDH) (Abcam; 1/2,500 for all). The following day, the membranes were washed in TBST five times, 5 min each before incubation for 1 hr at room temperature with specific antirabbit or antimouse secondary antibodies (Bio‐Rad Laboratories). Membranes were revealed with ECL chemiluminescent substrate (Bio‐Rad Laboratories, Inc.) and signals detected by an imaging system equipped with a CCD camera (Omega Lum G, Aplegen, Gel Company, SF, USA). Signal intensities of bands in the immunoblots were quantified using ImageJ analysis software. Three western blots were performed for each protein and condition.

### 4.6 Assessment of plasma levels of Il‐6, CRP, TGF‐β, and BNP

Blood samples were collected, on the time of sacrifice, in EDTA tubes from control and treated rats. Plasma samples were obtained by centrifugation at 4,500 rpm for 10 min and then aliquoted and stored at −80 ˚C until analysis. All plasma parameters measurements were done by the ELISA technique: TGF‐β (Rat) kit, IL‐6 (Rat) kit, CRP (Rat) kit, and BNP‐32 Rat kit (Abcam) according to the manufacturer's protocols.

### 4.7 Statistical analysis

Results are presented as the mean ± *SEM* for the number of samples indicated in the figure legends. One‐way ANOVA or two‐way ANOVA was used to test for significance between groups. Student–Newman–Keuls post hoc test was applied for multiple pairwise comparisons. Statistics were analyzed using SigmaPlot Software (version 12.5). Significance was set below 0.05 for all analysis.

## CONFLICT OF INTEREST

The research was conducted in the absence of any commercial or financial relationships that could be construed as a potential conflict of interest.

## AUTHORS’ CONTRIBUTIONS

SC, JFF, and NF designed the research, performed experiments, analyzed the data, and drafted the manuscript. JH and YS performed experiments and helped for data analysis. PB and NL helped for data acquisition. RGM helped in designing the research and reviewed the manuscript. All authors discussed the results and approved the final version of the manuscript.
