# Supplementary figures and images for: Long‐term intake of phenolic compounds attenuates age‐related cardiac remodeling
Source: Aging Cell. 2019 Jan 24;18(2):e12894. doi: 10.1111/acel.12894 (PMC6413651; doi:10.1111/acel.12894)

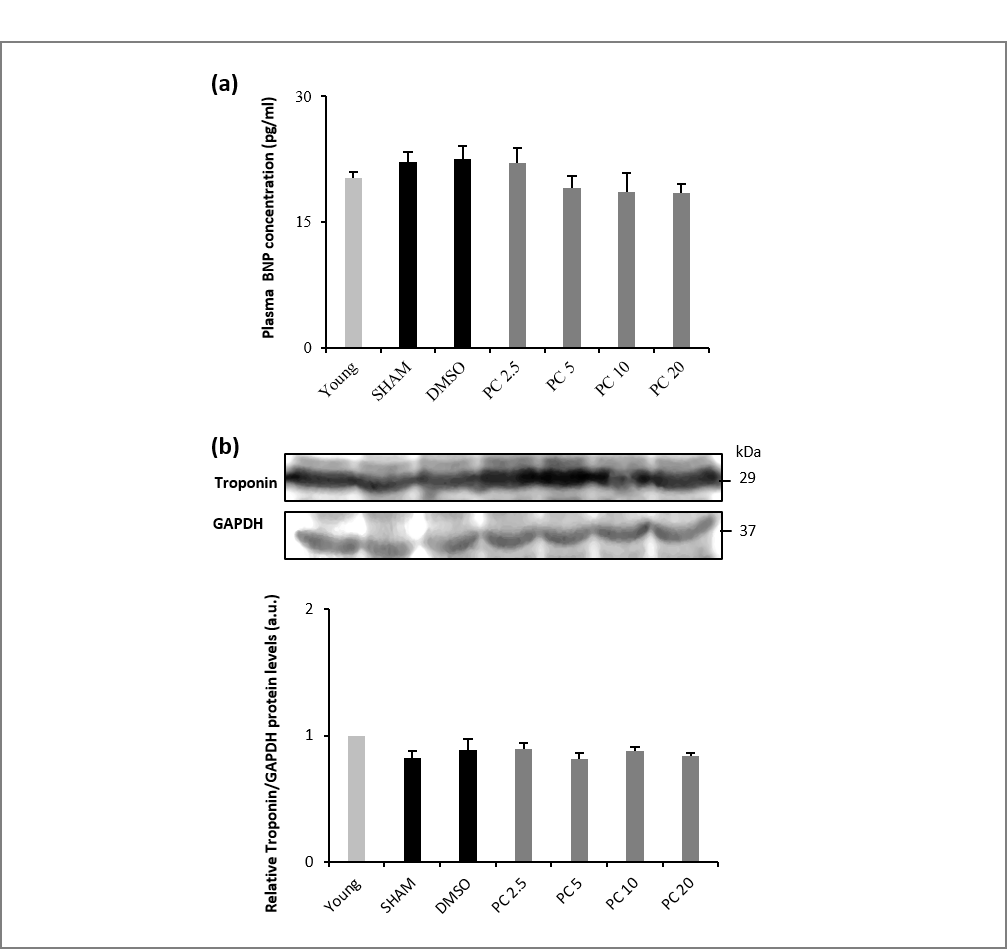

Supplement: Supplementary file 1 [file ACEL-18-e12894-s001.tif]
