# Supplementary material for: Long‐term intake of phenolic compounds attenuates age‐related cardiac remodeling
Source: Aging Cell. 2019 Jan 24;18(2):e12894. doi: 10.1111/acel.12894 (PMC6413651; doi:10.1111/acel.12894)
Supplement: Supplementary file 2 [file ACEL-18-e12894-s002.docx]

**Supplemental figures legends**

**Supplemental figure 1: Absence of heart failure in all control and experimental groups.** (a) BNP plasma levels in all groups of animals (n=6-8 per group). (b) Representative Western blot (left) and histograms analyses (right) for troponin in rat cardiac tissue, normalized to GAPDH (in arbitrary units, a.u.) (n=3 for each protein and condition). PC x = phenolic compounds at x mg/kg/day.
